# Diminished LAG3^+^ B cells correlate with exacerbated rheumatoid arthritis

**DOI:** 10.1080/07853890.2023.2208373

**Published:** 2023-05-04

**Authors:** Suiyuan Hu, Yuting Tao, Fanlei Hu, Xu Liu

**Affiliations:** a Department of Rheumatology and Immunology, Peking University People’s Hospital, Beijing Key Laboratory for Rheumatism Mechanism and Immune Diagnosis (BZ0135), Beijing, China; b State Key Laboratory of Natural and Biomimetic Drugs, School of Pharmaceutical Sciences, Peking University, Beijing, China; c Department of Integration of Chinese and Western Medicine, School of Basic Medical Sciences, Peking University, Beijing, China

**Keywords:** Immunotherapy, LAG3, rheumatoid arthritis, regulatory B cells

## Abstract

**Background:**

Lymphocyte activation gene-3 (LAG3) positive B cells have been identified as a novel regulatory B cell subset, while the role of LAG3^+^ B cells in the pathogenesis of rheumatoid arthritis (RA) remains elusive.

**Materials and methods:**

Peripheral blood mononuclear cells (PBMCs) from RA, osteoarthritis (OA) patients and healthy volunteers were collected for flow cytometry staining of LAG3^+^ B cells. Their correlation with RA patient clinical and immunological features were analyzed. Moreover, the frequencies of LAG3^+^ B cells in collagen-induced arthritis (CIA) mice and naive mice were also detected.

**Results:**

A significant decrease of LAG3^+^ B cells was observed in RA patients as compared with healthy individuals and OA patients. Notably, the frequencies of LAG3^+^ B cells were negatively correlated with tender joint count (r = −0.4301, *p* = .0157) and DAS28-ESR (r = −0.4018, *p* = .025) in RA patients. In CIA mice, LAG3^+^ B cell frequencies were also decreased and negatively correlated with the CIA arthritis score.

**Conclusions:**

Impairment of LAG3^+^ B cells potentially contributes to RA development. Reconstituting LAG3^+^ B cells might provide novel therapeutic strategies for the persistent disease.

**Key messages**
- LAG3^+^ B cells have been identified as a novel regulatory B cell subset. However, its role in the pathogenesis of RA remains unknown.
- This study revealed the decreased frequency of LAG3^+^ B cells in RA patients. Notably, LAG3^+^ B cells were negatively correlated with RA disease activity including the tender joint count and DAS28-ESR.
- In CIA mice, LAG3^+^ B cell frequencies were also decreased and negatively correlated with the CIA arthritis score.
- Reconstitution of LAG3^+^ B cells might provide novel therapeutic strategies for disease perpetuation.

## Introduction

Rheumatoid arthritis (RA) is a systemic autoimmune disease characterized by symmetrical polyarthritis, impairing bone and cartilage. Without early diagnosis or appropriate treatment, joint swelling and pain can turn into deformity and disability, which brings about individual and social burdens.

B cells play a vital role in the pathogenesis of RA [1]. Naive B cells are activated and differentiate into plasma cells which produce autoantibodies like rheumatoid factor and anti-citrullinated protein antibody (ACPA). These autoantibodies form immune complexes (IC) and trigger perpetual inflammation. Additionally, B cells can produce proinflammatory factors such as IL-6, which drives autoimmune reactions [2]. Thus, various B-cell-targeting drugs have been developed, such as rituximab, a chimeric murine/human monoclonal CD20 antibody. While its effectiveness has been verified by a phase III clinical trial, only 30% of RA patients treated with rituximab showed an American College of Rheumatology 50% improvement criteria (ACR50) response at 24 weeks [3]. One of the underlying reasons might be the reduction of regulatory B (Breg) cells in general B cell depletion therapy.

Different phenotypes of Breg cells have been demonstrated to exert immunosuppressive functions in RA, including B10 cells and transitional 2 marginal-zone precursor (T2-MZP) Breg cells. Despite their different phenotypes, Breg cells negatively regulate immunity *via* the production of inhibitory cytokines [4]. IL-10-producing Breg cells are demonstrated to ameliorate collagen-induced arthritis (CIA) in mice, indicating they might emerge as important players in RA intervention [5].

Lymphocyte activation gene-3 (LAG3), also named CD223, a member of the inhibitory receptor family, has achieved extensive attention and has been regarded as the next promising immune checkpoint target recently. It is widely expressed on immune cells such as CD4^+^ T cells, CD8^+^ T cells, Tregs, B cells, and NK cells [6]. LAG3 is structurally similar to CD4, while it can interact with major histocompatibility complex (MHC) II with a 100-fold affinity that of CD4 and significantly inhibit CD4^+^ T cell proliferation and activation [7,8]. A study showed LAG3 antibodies block Treg activity both *in vitro* and *in vivo*, and the regulatory activity of Tregs from LAG3^−/−^ mice was impaired, suggesting the indispensable role of LAG3 in Treg function [9].

The intricate relationship between LAG3 and T cells in the tumor environment and autoimmunity has been widely discussed. Nowadays, LAG3 on B cells is receiving increasing attention. Recently, LAG3 was demonstrated to be able to identify a novel subset of natural plasma cells. The subset of LAG3^+^CD138^hi^ cells was detected in naive mice and can rapidly produce IL-10 after infection, indicating its underlying role in preventing immune overreaction [10]. Another subset of Breg cells characterized by IL-27 production was shown to upregulate LAG3 expression and inhibit Th17 responses, thus ameliorating experimental autoimmune encephalomyelitis (EAE) [11].

Considering the negative regulatory role of LAG3^+^ B cells in autoimmune diseases, we hypothesized that LAG3^+^ B cells were downregulated in RA patients. In this study, we characterized the decreased frequency of LAG3^+^ B cells in RA patients as well as in the CIA model. RA disease activity was negatively correlated with the frequency of LAG3^+^ B cells. Our study revealed the possible capacity of LAG3^+^ B cells to maintain immune tolerance in autoimmune diseases, demonstrating their potential in RA activity evaluation and disease therapy.

## Materials and methods

### Study population and serum samples

Fresh peripheral blood (PB) samples from 46 RA patients, 20 OA patients and 57 healthy volunteers were obtained between 2018 and 2019 at the Department of Rheumatology and Immunology, Peking University People’s Hospital, Beijing, China. All RA patients fulfilled the 2010 American College of Rheumatology (ACR) and European League Against Rheumatism (EULAR) criteria for RA, while all OA patients met the ACR 1995 classification criteria. The study was approved by the Research Ethics Committee at Peking University People’s Hospital, Beijing, China. All patients and healthy donors gave informed consent.

### Mice

Six- to eight-week-old male DBA/1 mice were obtained from Huafukang Bioscience Company (Beijing, China). All mice were housed in specific pathogen-free environment under controlled conditions (22 °C ambient temperature, 40% humidity). All animal procedures complied with relevant ethical regulations for animal testing and research, and were approved by the institutional animal care and use committee (IACUC) of Peking University People’s Hospital and Virginia Commonwealth University.

### CIA models

For arthritis induction, DBA/1 mice were immunized at the base of the tail with the emulsion of 200 μg bovine type II collagen in complete Freund adjuvant (CFA) on day 1. On day 21, the emulsion of 100 μg bovine type II collagen in incomplete Freund adjuvant (IFA) was injected at the same site as disease induction. The severity of arthritis was scored based on the level of inflammation in each of the four paws and recorded as one of four grades: 0, normal; 1, erythema and swelling of one or several digits; 2, erythema and moderate swelling extending from the ankle to the mid-foot (tarsals); 3, erythema and severe swelling extending from the ankle to the metatarsal joints; and 4, complete erythema and swelling encompassing the ankle, foot, and digits, resulting in deformity and/or ankyloses. The scores of all four limbs were summed, yielding a total score of 0–16 per mouse.

### Flow cytometry

To detect LAG3 expression in human B cell subsets, PBMCs from healthy individuals, RA or OA patients were stained with anti-LAG3-BV650. Fluorescence-minus-one (FMO) controls were used as negative control. PBMCs were stained with anti-CD3-PerCP-eFluo710, anti-CD19-APC-Fire750, anti-CD20-PE-CY7, anti-CD24-FITC, anti-CD27-APC, anti-CD43-PE and anti-IgD-Pacific-Blue at room temperature for 30 min, washed with PBS and then re-suspended and fixed with 1.5% formaldehyde for 2 min under room temperature. Cells were analyzed on FACSAria II flow cytometer.

To detect LAG3 expression in mouse B cell subsets, splenocytes from naive mice and CIA mice were stained with anti-LAG3-BV650, anti-CD19-PerCP-CY5.5, anti-CD80-FITC, anti-CD86-PE, anti-CD138-APC, anti-CD69-PE1CY7. Cells were stained at room temperature for 30 min, washed with PBS and then re-suspended and fixed with 1.5% formaldehyde for 2 min under room temperature. Cells were analyzed on FACS Arial II flow cytometer. Gating strategies are presented in Supplementary Figure 1.

### Statistics

Statistical analyses were performed using the statistical software programs GraphPad Prism 8 (GraphPad Software Inc., San Diego, CA) and SPSS 25 (SPSS Inc.). Differences between various groups were evaluated by the unpaired t test, Mann-Whitney test, Friedman test, Kruskal-Wallis test, Pearson correlation test or Spearman’s correlation test. All analyses with *p* value <.05 were considered statistically significant (*p < .05, **p < .01, ***p < .001, N.S., not significant).

## Results

### Expression of LAG3 in healthy individual B cell subsets

**Figure 1.**
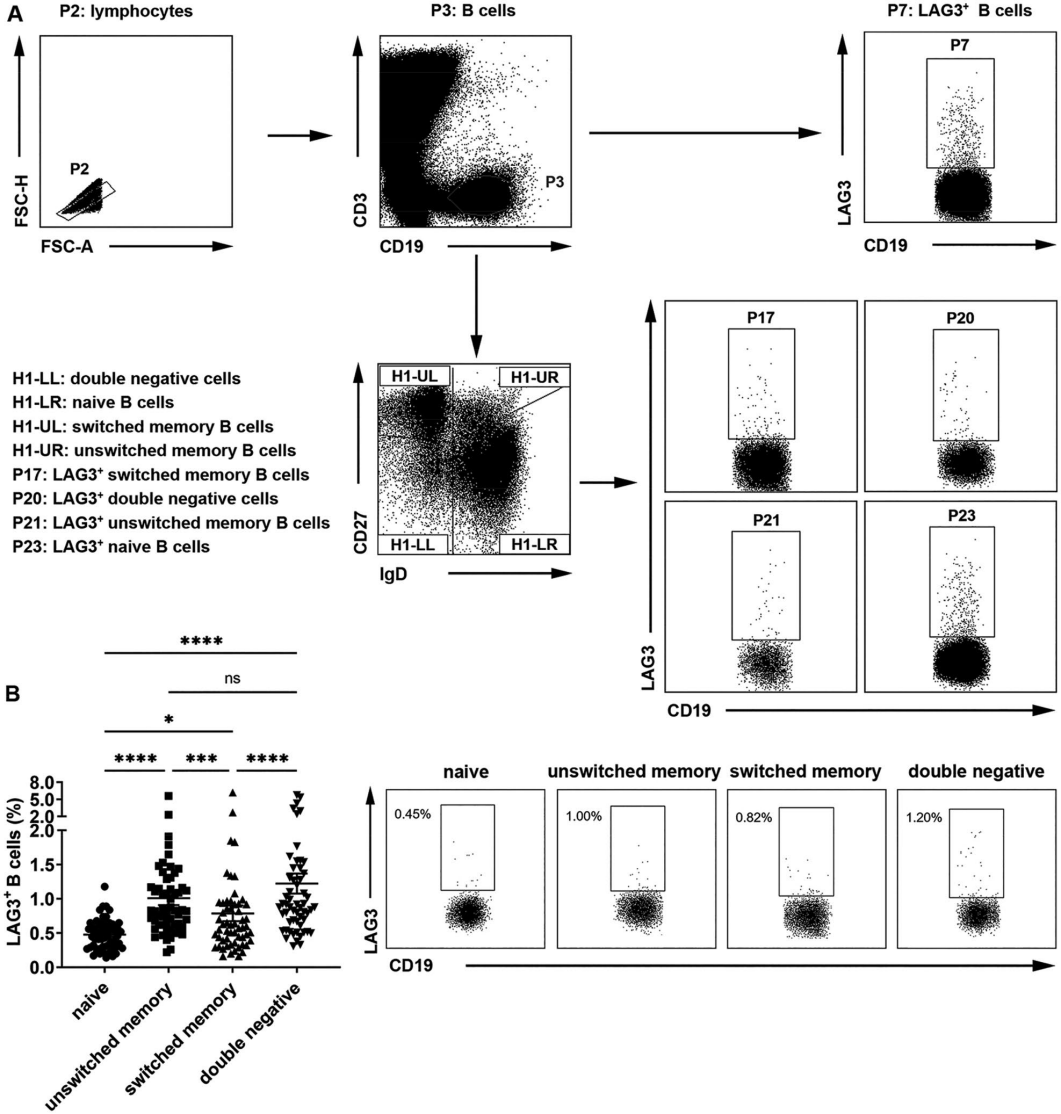
Different fractions of LAG3^+^ B cells in healthy donor B cell subsets. (A) Gating strategies of peripheral blood mononuclear cells from healthy individuals, RA or OA patients. CD3^−^CD19^+^ B cells (P3) were sorted into CD27^−^IgD^−^ double negative cells (H1-LL), CD27^−^IgD^+^ naive B cells (H1-LR), CD27^+^IgD^-^ switched memory B cells (H1-UL) and CD27^+^IgD^+^ unswitched memory B cells (H1-UR). LAG3^+^ cells in B cells and all B cell subsets were selected (P7, P17, P20, P21, P23). (B) Flow cytometric analysis of the frequencies of LAG3^+^ B cells from healthy individuals (*n* = 57). Statistical results and the representative flow charts were shown. Data were presented as mean ± SEM. Results were representative of fifteen independent experiments. *p < .05, ***p < .001, ****p < .0001, ns, not significant (Friedman test followed by Dunn’s multiple comparison test).

We first detected the frequencies of LAG3^+^ B cells in healthy individuals. Peripheral blood B cells were classified into four populations based on IgD and CD27 expression on cell surfaces: CD19^+^CD27^−^IgD^+^ naive B cells, CD19^+^CD27^+^IgD^+^ unswitched memory B cells, CD19^+^CD27^+^IgD^-^ switched memory B cells and CD19^+^CD27^−^IgD^−^ double negative B cells (Figure 1(A)). Previous studies demonstrated CD27^+^IgD^+^ unswitched memory B cells were innate-like B cells and could produce protective natural IgM in RA [12], whereas CD27^−^IgD^−^ double negative B cells exacerbated systemic lupus erythematosus (SLE) patients and were linked to renal impairment [13]. Our results showed that, compared with naive B cells which exhibited the lowest fraction of LAG3^+^ B cells, unswitched memory B cells and double negative B cells demonstrated relatively high frequencies of LAG3^+^ B cells (Figure 1(B)). These results showed that there was increased LAG3^+^ B cell frequency on both effector and inhibitory B cells in healthy people.

### Decreased frequencies of LAG3^+^ B cells in RA patients

Given the immunoregulatory functions of LAG3, we next sought to test whether LAG3^+^ B cells decreased in RA patients, thus impairing the immunosuppressive functions of B cells and promoting disease perpetuation. Demographic characteristics of RA patients and controls were shown in Table 1.

**Table 1.** Demographic characteristics of RA patients and controls.

Characteristics	RA (*n* = 46)	OA (*n* = 20)	HC (*n* = 57)
Age (years)	58.79 ± 12.00	53.59 ± 9.875	51.44 ± 19.87
Sex (female%)	78.26	88.23	92.86
Duration (years)	11.02 ± 10.30		
Tender joint count	9.774 ± 10.50		
Swollen joint count	6.097 ± 7.956		
DAS28 (of 28 joints)	4.812 ± 2.019		
ESR (mm/h)	40.95 ± 33.90		
CRP (mg/l)	28.70 ± 34.54		
RF (+) (%)	24/31 (77.42)		
Anti-CCP antibody (+) (%)	23/31 (74.19)		
IgG (g/l)	13.47 ± 4.829		
IgA (g/l)	2.777 ± 1.443		
IgM (g/l)	1.121 ± 0.4820		
Medications (*n* = 36)			
DMARDs naive^a^	*n* = 14 (38.89%)		
csDMARDs monotherapy^b^	*n* = 11 (30.55%)		
csDMARDs combination therapy^c^	*n* = 5 (13.89%)		
bDMARDs + csDMARDs^d^	*n* = 6 (16.67%)		

*Notes:* Detailed therapies of 10 RA patients were unavailable and not shown in the table. Numerical data were presented as mean ± SD and the range of statistics. RA: rheumatoid arthritis; OA: osteoarthritis; HC: healthy control; ESR: erythrocyte sedimentation rate; CRP: C-reactive protein; RF: rheumatoid factor; anti-CCP antibody: anti-cyclic citrullinated peptide antibody; IgG: immune globulin G; IgA: immune globulin A; IgM: immune globulin M; DMARDs: disease-modifying antirheumatic drugs; csDMARDs: conventional synthetic; bDMARDs: biological DMARDs.

^a^The DMARDs naive group included patients who had not received DMARDs or had been on DMARDs for less than two months.

^b^The csDMARDs monotherapy group included patients who had been on only one kind of csDMARDs for more than two months and had not used bDMARDs.

^c^The csDMARDs combination therapy group included patients who had used more than one kind of csDMARDs for over two months and had not used bDMARDs.

^d^The bDMARDs + csDMARDs group has been on bDMARDs and csDMARDs over two months.

**Figure 2.**
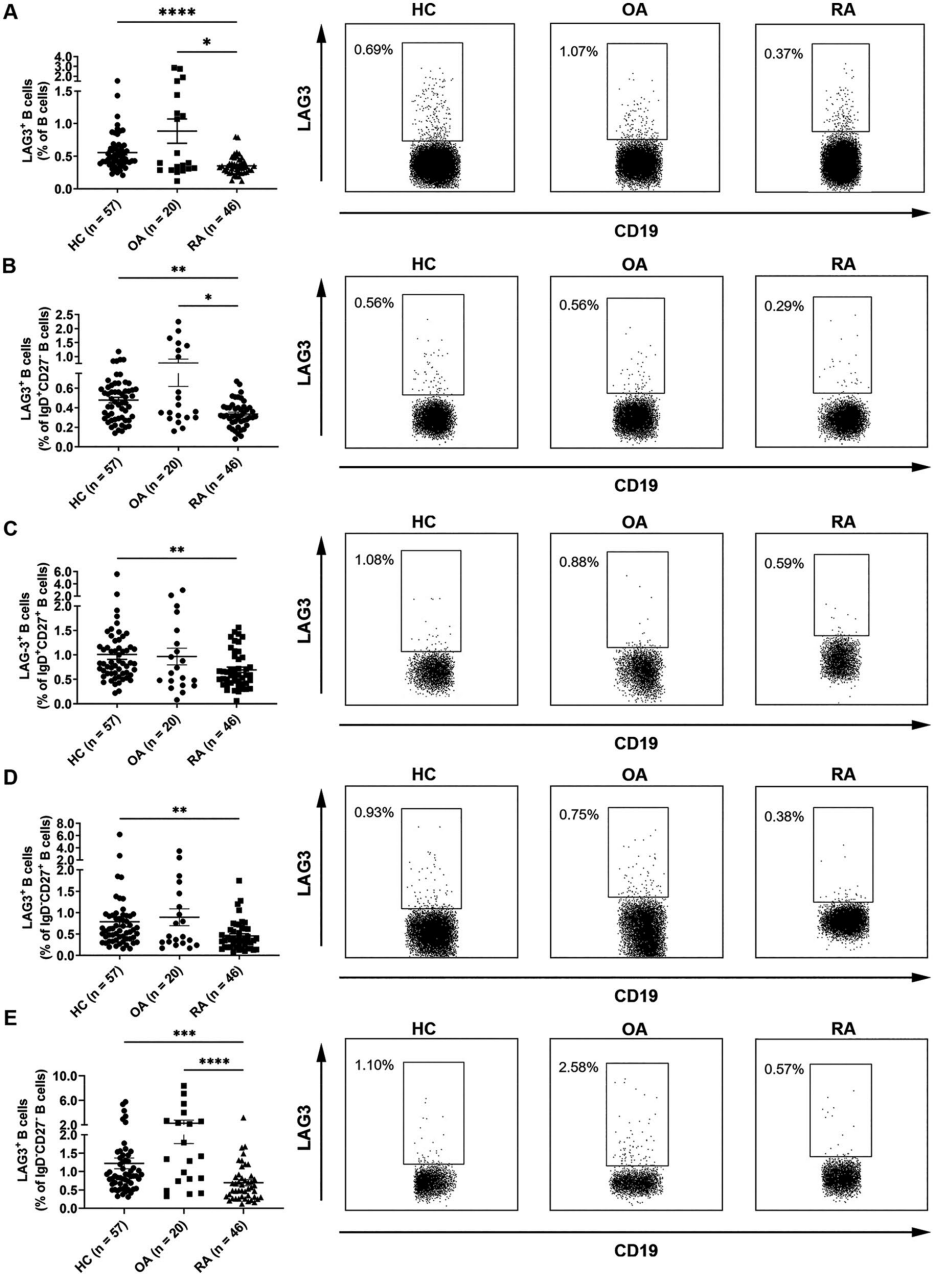
Decreased frequencies of LAG3^+^ B cells in RA patients. Flow cytometric analysis of the frequencies of LAG3^+^ B cells in the peripheral blood B cells from healthy individuals (*n* = 57), osteoarthritis patients (*n* = 20) and rheumatoid arthritis patients (*n* = 46). The frequency of LAG3^+^ B cells in peripheral blood B cells (A), naive B cells (CD27^−^IgD^+^ B cells) (B), unswitched memory B cells (CD27^+^IgD^+^ B cells) (C), switched memory B cells (CD27^+^IgD^-^ B cells) (D) and double negative B cells (CD27^−^IgD^−^ B cells) (E) from RA patients were down-regulated as compared with HC and OA control. Data were presented as mean ± SEM. Results were representative of four independent experiments. *p <.05, **p <.001, ***p <.001, ****p <.0001, NS: not significant; HC: healthy control; OA: osteoarthritis; RA: rheumatoid arthritis (Kruskal–Wallis test followed by Dunn’s multiple comparison test).

As shown in Figure 2, under RA circumstances, LAG3^+^ B cell frequencies in peripheral blood total B cells (**A**), naive B cells (**B**), unswitched memory B cells (**C**), switched memory B cells (**D**) and double negative B cells (**E**) were all down-regulated as compared with healthy individuals and OA patients, despite the reduction in unswitched memory B cells and switched memory B cells was not statistically significant. Additionally, the frequency of LAG3^+^ B cells in lymphocytes was also significantly decreased in RA patients (Table 2).

**Table 2.** The frequency of LAG3^+^ B cells in lymphocytes of HC, OA and RA (‰0).

Statistics	HC (*n* = 57)	OA (*n* = 20)	RA (*n* = 46)
Mean	2.741	4.611	1.988
Std. deviation	1.361	4.492	1.298
Std. error	0.1802	1.0040	0.1914
*p* Value	0.8458 ^ns^	0.003400**	0.008400**
	(vs. OA)	(vs. RA)	(vs. HC)

*Note:* HC: healthy control; OA: osteoarthritis; RA: rheumatoid arthritis; ns: not significant.

**p < .01.

These results demonstrated that LAG3^+^ B cells were diminished in lymphocytes as well as all the subsets of B cells under RA circumstances, indicating LAG3^+^ B cells might be able to intervene in RA and the reduction of LAG3^+^ B cells might contribute to RA pathogenesis.

### Correlation of LAG3^+^ B cells with clinical and immunological features of RA patients

Then we analyzed the correlation between LAG3^+^ B cells and clinical parameters in RA patients (Table 3).

**Table 3.** Correlation of LAG3^+^ B cell frequencies with clinical and laboratory features of RA patients.

Features	R	*p*
Age	0.0104	.947
Duration	0.0588	.726
ESR	−0.213	.182
Anti-CCP antibody	−0.0297	.874
RF	0.107	.565
CRP	−0.239	.161
DAS28-ESR	**−0.402**	.0250*
IgA	−0.0866	.655
IgM	0.0111	.954
IgG	−0.177	.358
Swollen joint counts	−0.289	.115
Tender joint counts	**−0.430**	.0157*

*Note:* ESR: erythrocyte sedimentation rate; anti-CCP antibody: anti-cyclic citrullinated peptide antibody; RF: rheumatoid factor; CRP: C-reaction protein. *p < 0.05.

**Figure 3.**
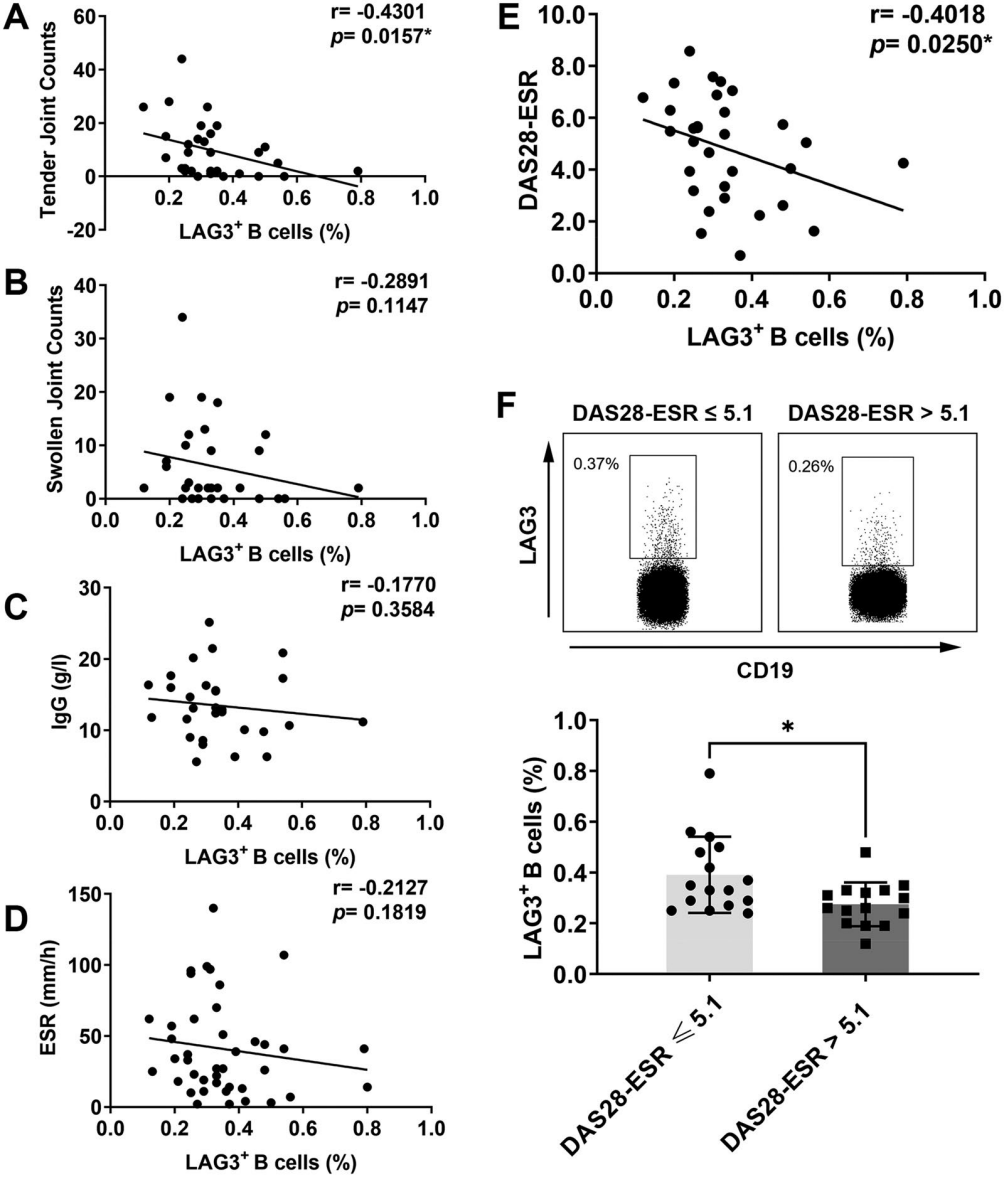
Correlation of LAG3^+^ B cells with clinical and immunological features of RA patients. The correlation of LAG3^+^ B cell frequencies with tender joint count (A), swollen joint count (B), concentrations of IgG in serum (C) and ESR in serum (D) was analyzed. The frequencies of LAG3^+^ B cells were negatively correlated with RA manifestations aforementioned. (E) Correlation analysis of the frequency of LAG3^+^ B cells with DAS28-ESR score. (F) The frequencies of LAG3^+^ B cells were compared between two groups (unpaired *t* test, *p <.05). Notably, in contrast to patients with DAS28-ESR ≤ 5.1, high disease activity group including patients with DAS28-ESR > 5.1 showed significantly lower fractions of LAG3^+^ B cells. The frequencies of LAG3^+^ B cells from RA patients (*n* = 46) were analyzed by flow cytometry, and the representative flow charts from two independent experiments were shown. Statistics analysis was performed using Spearman’s rank correlation test.

As for clinical features, tender joint count showed a significant negative correlation with the frequency of LAG3^+^ B cells (Figure 3(A)). Swollen joint count also negatively correlated with the fraction of LAG3^+^ B cells, but the difference was not statistically significant (Figure 3(B)). Patients of greater age didn’t demonstrate lower frequencies of LAG3^+^ B cells than younger patients. Additionally, no significant correlation was found between LAG3^+^ B cell frequencies and the disease duration of RA patients. As for laboratory parameters, no significant correlation was found between the fraction of LAG3^+^ B cells and serum IgG (Figure 3(C)), IgA, IgM or the levels of ESR (Figure 3(D)), CRP. Autoantibodies were not correlated with the frequency of LAG3^+^ B cells either, including anti-cyclic citrullinated peptide (anti-CCP) antibody and RF. Then we explored the relationship between LAG3^+^ B cells and disease activity demonstrated by DAS28-ESR. As shown in Figure 3(E), DAS28-ESR scores negatively correlated with the frequencies of LAG3^+^ B cells in RA patients. Moreover, as shown in Figure 3(F), RA patients with high disease activity (DAS28 > 5.1) displayed lower frequencies of LAG3^+^ B cells than patients with non-high disease activity (DAS28 ≤ 5.1). Overall, the fraction of LAG3^+^ B cells was negatively correlated with tender joint count and DAS28 score. This result suggested that diminished LAG3^+^ B cells correlated with RA exacerbation, indicating LAG3^+^ B cells might play a protective role in RA progression.

### Diminished LAG3^+^ B cells in CIA mice

**Figure 4.**
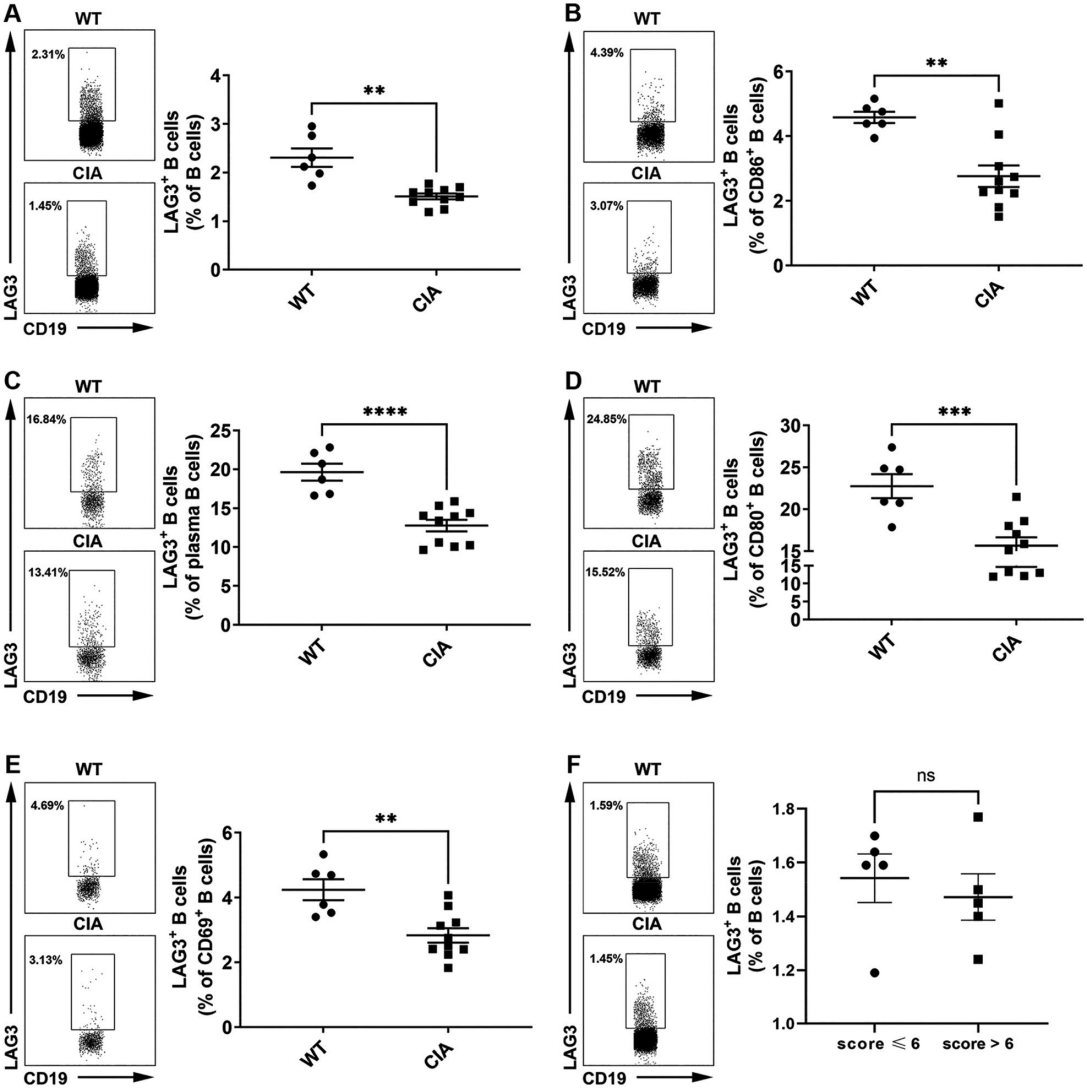
Diminished LAG3^+^ B cells in collagen-induced arthritis (CIA) mice. The frequencies of LAG3^+^ B cells (A), LAG3^+^CD86^+^ B cells (B), LAG3^+^ plasma B cells (C), LAG3^+^CD80^+^ B cells (D) and LAG3^+^CD69^+^ B cells (E) were significantly diminished in CIA mice as compared with naive mice (Kruskal–Wallis test followed by Dunn’s multiple comparison test). The frequencies of LAG3^+^ B cells from naive mice (*n* = 6) and CIA mice (*n* = 10) were analyzed by flow cytometry. Statistical results and the representative flow charts were shown. CIA mice were divided into high disease activity group (score > 6) and non-high disease activity group (score ≤ 6). High disease activity group showed lower frequencies of LAG3^+^ B cells in comparison with non-high disease activity group, but the disparity was not significant (*p* =.5906) (F). Unpaired *t* test was performed to analyze statistics. Results were representative of seven independent experiments.

To further confirm the diminished LAG3^+^ B cells under RA circumstances *in vivo*, we further examined the frequencies of LAG3^+^ B cells in CIA mice. Our results showed that the frequencies of LAG3^+^ B cells (Figure 4(A)), including LAG3^+^ CD86^+^ B cells (Figure 4(B)), LAG3^+^ plasma B cells (Figure 4(C)), LAG3^+^ CD80^+^ B cells (Figure 4(D)) and LAG3^+^ CD69^+^ B cells (Figure 4(E)), significantly decreased in CIA mice as compared with control mice. Notably, plasma B cells and CD80^+^ B cells demonstrated high fractions of LAG3^+^ B cells. To identify the correlation between disease activity and LAG3^+^ B cell frequencies, CIA mice were divided into a high disease activity group (score > 6) and a non-high disease activity group (score ≤ 6). Compared to non-high disease activity group, LAG3^+^ B cell frequencies decreased in high disease activity group (Figure 4(F)). However, the disparity was not statistically significant (*p* = .5906). These results exhibited the downregulation of LAG3^+^ B cells in CIA mice especially in activated B cells, which was consistent with our results aforementioned.

## Discussion

Previous studies showed that LAG3, as a member of the inhibitory receptor family, played an important role in maintaining immune tolerance. In this study, we described the decreased frequency of LAG3^+^ B cells under RA circumstances. Functionally, we also demonstrated that LAG3^+^ B cells were negatively correlated with RA disease activity. The reduction of LAG3^+^ B cell frequencies was also observed in CIA *in vivo*. Our results provide evidence that LAG3^+^ B cells decreased as disease progressed and might exert immunosuppressive functions in RA intervention.

The correlation of Breg cells with RA has been studied intensely [14–16]. IL-10-producing regulatory B10 cells were able to ameliorate CIA [5]. An early study demonstrated an increased frequency of B10 in RA and other autoimmune diseases as compared with healthy controls [14]. However, recent research reported regulatory B10 cells were downregulated or functionally impaired in patients with RA and were negatively correlated with disease activity [15,16]. Recently, LAG3^+^ B cells have been identified as a novel immunosuppressive regulatory B cell subset [10], while the role of LAG3^+^ B cells in RA has not been discussed yet. In this study, we first reported diminished LAG3^+^ B cells in RA and their correlation with aggravated RA, and these results were verified in the CIA mouse model.

Just like other inhibitory receptors, LAG3 limits the over-activation of pathogenic self-reactive T cells and protects tissues and organs from autoimmune attack under normal circumstances [17–19]. For LAG3 and CD4 have similar structures, LAG3 can effectively interact with MHC II and inhibit CD4^+^ T cells expansion [7], which may help to explain the immunosuppressive function of LAG3^+^ B cells. Also, considering LAG3^+^ B cells define the major population of IL-10-expressing B cells in infected mice [10], these cells might regulate immune function *via* the production of inhibitory cytokines such as IL-10 and TGF-β. However, how LAG3^+^ B cells participate in RA pathogenesis requires further exploration. The recombinant cytokine F8-IL10 shows promising results in inhibiting CIA progression [20,21], making it tempting to speculate that LAG3^+^ B cells intervene in RA by anti-inflammatory cytokine production and subsequent T cell inhibition.

B10 cells are able to convert into RANKL-producing cells, which significantly exacerbate bone erosion and are positively correlated with disease activities [22]. This pathogenic conversion might partially explain the diminished LAG3^+^ B cells in RA patients. The decreased frequency of LAG3^+^ B cells might contribute to increased apoptosis, which was seen in memory Breg cells in patients with systemic sclerosis [23,24]. Generally, the mechanism behind the significant downregulation of LAG3^+^ B cell frequencies in RA remains unknown.

Increasing evidence has revealed the promising potency of regulatory cells in RA therapy. In the murine CIA model, adoptive transfer of LAG3^+^ Treg-of-B cells (Treg cells induced by B cells) showed pronounced alleviation of the joint severity as well as local and systemic inflammation [25]. As for Breg cells, animal experiments have shown that transferring regulatory B cells inhibits the progression of EAE [26] and SLE [27]. However, there have been no animal experiments or clinical trials targeting Breg cells in RA. Our study demonstrated the potential therapeutic role of LAG3^+^ B cells in RA patients, which requires further research.

One limitation of this study is that only frequencies of cells rather than absolute cell numbers were calculated and compared. Our results reflected decreased frequencies of LAG3^+^ B cells in RA patients and CIA mice. However, there might be a disparity in immune cell numbers between healthy controls and arthritis samples that cannot be shown in this study. In further exploration, the absolute count of cells is needed to confirm whether LAG3^+^ B cells diminish in RA patients and CIA mice.

Additionally, previous studies demonstrated that abatacept treatment targeting T-cell responses might modulate Bregs and LAG3^+^ Tregs in RA patients [28,29], indicating therapy given to RA patients might affect the level of LAG3^+^ B cells. In this study, the difference in LAG3^+^ B cells among patients treated with various medications was not observed. However, the impact of therapies on LAG3^+^ B cells has not been fully assessed, which requires further exploration.

Consistent with the aforementioned findings, in this study we showed that under RA circumstances, the frequency of LAG3^+^ B cells was reduced in B cells and negatively correlated with RA clinical features and disease activity, which was verified in the CIA mouse model. According to our study, searching for new reagents that promote LAG3^+^ B cells in RA patients or transferring LAG3^+^ B cells into RA patients might provide novel therapeutic strategies for RA.

## Conclusions

In summary, here we revealed diminished LAG3^+^ B cells in RA that might exacerbate the immune disorder and perpetuate the disease. Modulating the status of LAG3^+^ B cells might provide novel therapeutic strategies for RA.

## Supplementary Material

Supplemental Material

## Data Availability

The data that support the findings of this study are available from corresponding authors, FL. H. and X. L., upon reasonable request.
