# Supplementary material for: Diminished LAG3+ B cells correlate with exacerbated rheumatoid arthritis
Source: Ann Med. 2023 May 4;55(1):2208373. doi: 10.1080/07853890.2023.2208373 (PMC10165927; doi:10.1080/07853890.2023.2208373)
Supplement: Supplemental Material [file IANN_A_2208373_SM2582.doc]

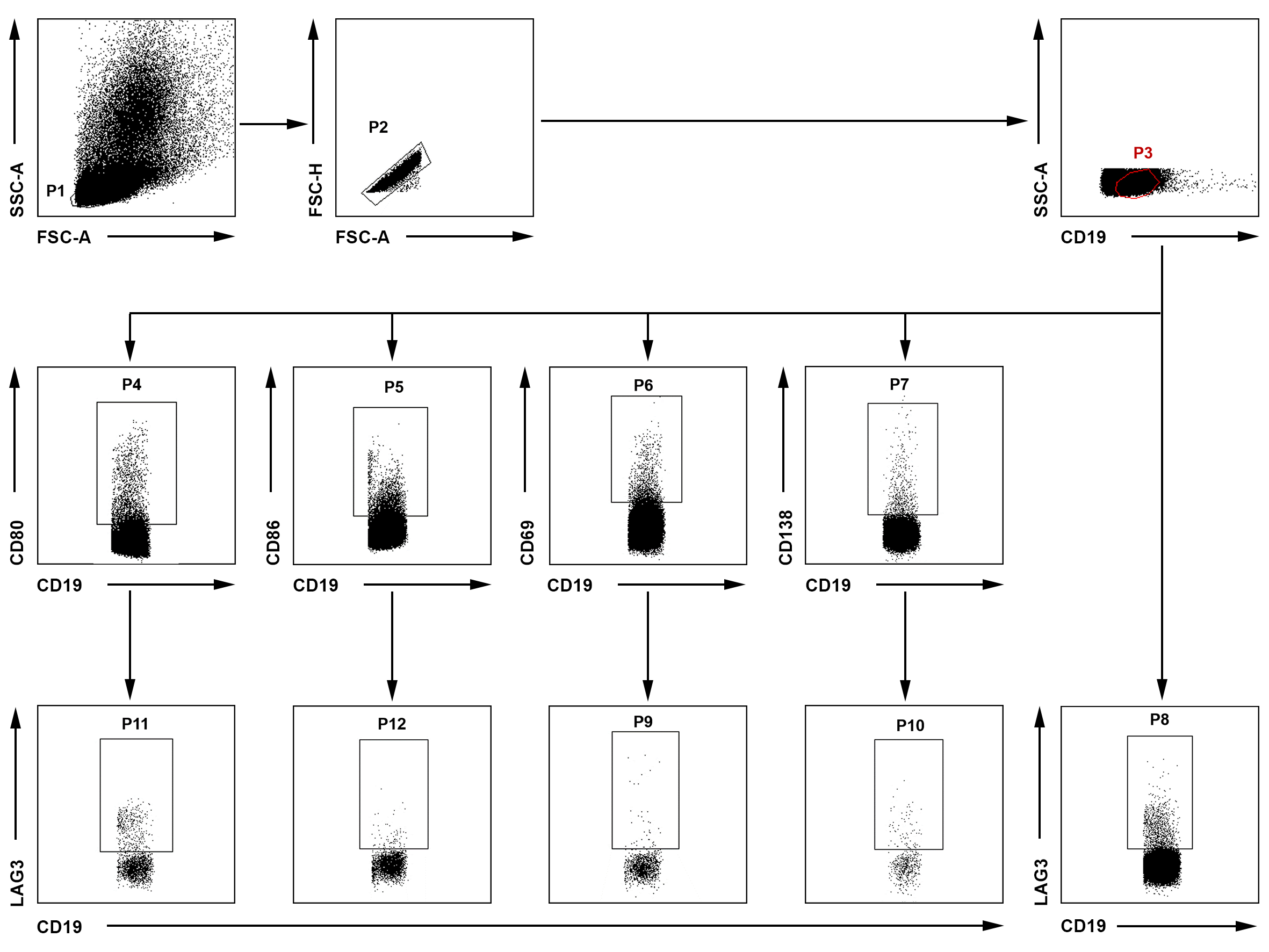


**Supplementary Figure 1** Gating strategies of splenocytes from naive mice and CIA mice. CD3^-^CD19^+^ B cells (P3) were sorted into CD80^+^ cells (P4), CD86^+^ B cells (P5), CD69^+^ activated B cells, CD138^+^ plasma B cells. LAG3^+^ cells in B cells and all B cell subsets were selected (P8, P9, P10, P11, P12).
